# Effectiveness and Safety of SGLT2 Inhibitors in Clinical Routine Treatment of Patients with Diabetes Mellitus Type 2

**DOI:** 10.3390/jcm10040571

**Published:** 2021-02-03

**Authors:** Maximilian Hopf, Christof Kloos, Gunter Wolf, Ulrich Alfons Müller, Nicolle Müller

**Affiliations:** 1 Department of Internal Medicine III, University Hospital Jena, 07747 Jena, Germany; Maximilian.hopf@web.de (M.H.); Christof.kloos@med.uni-jena.de (C.K.); gunter.wolf@med.uni-jena.de (G.W.); 2 Practice for Endocrinology and Diabetology, Centre for Ambulatory Medicine, Jena University Hospital, 07743 Jena, Germany; UA.Mueller@t-online.de

**Keywords:** clinical routine, diabetes mellitus, genital tract infections, SGLT2 inhibitors, treatment discontinuation

## Abstract

The aim of this study was to investigate the effectiveness of SGLT2 inhibitors with regard to metabolic parameters and patient safety under routine ambulatory conditions. Retrospective longitudinal study of 95 patients with type 2 diabetes (diabetes duration 13.3 y; HbA1c 8.9%; eGFR 80.1 mL/min) receiving SGLT-2-inhibitors. Metabolic control and adverse event profile were evaluated. The mean follow-up time was 1.2 ± 0.8 years. The following changes were observed: HbA1c −1.0% ± 1.9 (*p* < 0.001), eGFR −7.0 mL/min ± 13.3 (*p* < 0.001), albuminuria −23.9 mg/g creatinine ± 144.5 (*p* = 0.118), bodyweight −3.0 kg ± 5.8 (*p* < 0.001), systolic blood pressure −6 mmHg ± 22 (*p* = 0.01), diastolic blood pressure −2 mmHg ± 14 (*p* = 0.243). 53 participants continuously applied the therapy. Twenty-eight participants discontinued SGLT-2-inhibitors due to various reasons: 20 participants because of genital- or urinary tract infections. One for dysuria, seven due to reduced eGFR below 45 mL/min. This study showed a considerable reduction of HbA1c and a modest reduction of eGFR, bodyweight and systolic blood pressure under clinical routine conditions. Genital infections occurred markedly more often than in randomized controlled trials. To apply SGLT-2-inhibitors more safely in clinical routine individual risks for genital and urinary tract infections should be considered and re-evaluated during therapy.

## 1. Introduction

There are numerous placebo-controlled studies that have investigated the effect of sodium-glucose cotransporter-2 inhibitors (SGLT2 inhibitors) in people with type 2 diabetes (EMPAREG, CANVAS, CREDENCE, DECLARE) [1]. The aim of these studies was to investigate the safety of SGLT2 inhibitors with respect to major adverse cardiovascular and renal outcomes. A systematic review of the cardiovascular outcome trials shows a benefit on atherosclerotic major adverse cardiovascular events in patients with established atherosclerotic cardiovascular disease [1]. They also reduce the risk of hospitalization due to heart failure and reduce the progression of renal disease, regardless of pre-existing heart failure or atherosclerotic cardiovascular disease [2]. Clinical parameters of interest are HbA1c, bodyweight, systolic and diastolic blood pressure, albuminuria and the occurrence of hypoglycemia. SGLT2 inhibitors are known to lower the HbA1c, bodyweight and systolic blood pressure [3]. After initial lowering of the estimated glomerular filtration rate (eGFR) because of reducing hyperfiltration, long-term renal function is preserved better and reduces less with SGLT2 inhibitor treatment [4]. Based on these placebo-controlled studies, some guidelines now recommend the use of SGLT2 inhibitors as first-line or second-line therapy after metformin in patients with cardiovascular disease, diabetic nephropathy, heart failure or corresponding risks [5]. Frequent adverse events of these drugs are genital and urinary tract infections [6]. In the randomized controlled trials, genital infection occurs with an event rate below 7% (EMPAREG, DECLARE) [7,8]. Data of routine care is still rare. This study was undertaken to provide data from routine care on the effectiveness regarding metabolic parameters and blood pressures, and the frequency of adverse effects.

## 2. Materials and Methods

This trial was designed as a retrospective longitudinal study. Data were collected in the department of Endocrinology and Metabolic diseases of the University Hospital Jena. All people with diabetes type 2 in the period between 1 January 2015 and 31 July 2017 receiving SGLT2 inhibitors as monotherapy or in combination with other antidiabetic drugs were included. Empagliflozin (25 mg daily) and dapagliflozin (10 mg daily) were the agents used in this study. The minimal treatment duration for inclusion in the analysis was one month. Laboratory and clinical data were collected from the electronic patients record EMIL™. If the SGLT2 inhibitor was initially prescribed by the general practitioner, necessary baseline parameters were requested from the general practitioner with written consent of the patients. We excluded persons with missing follow-up data or duration of treatment less than one month and without a valid declaration of agreement. Follow-up data were obtained at the end of the observation period or if SGLT2 inhibitors were discontinued. 

Informed Consent Statement: All procedures followed were in accordance with the ethical standards of the committee on human experimentation of the study institutions and German national standards as well as with the Helsinki Declaration of 1975, as revised in 2008. Ethical review and approval were waived for this study, due to the retrospective design of the study. Only a retrospective analysis of data collected during routine visits was carried out. There was no study-specific intervention.

### 2.1. Outcomes

Outcome parameters were HbA1c, bodyweight, blood pressure, eGFR, albuminuria, hypoglycemia and the adverse effect profile. We determined clinical and laboratory data and assessed metabolic control before start of treatment with SGLT2 inhibitor and at the end of observation. HbA1c was measured using high-performance liquid chromatography (TOSOH-Glykohaemoglobin-Analyzer HLC-723 GhbV, TOSOH CORPORATION, Tokyo, Japan) with a normal range of 5.0–6.2%. HbA1c was adjusted according to the mean normal value of healthy people (5.05%, 32 mmol/mol) of the Diabetes Control and Complications Trial (DCCT). Albuminuria was measured in milligram albumin per gram creatinine in the second morning spontaneous urine sample (mg/g Crea). Antihypertensive therapy was recorded in detail to evaluate the effect of SGLT-2-inhibitors on blood pressure. Classes of antihypertensive drugs such as ACE-inhibitors or angiotensin II receptor blockers (ARBs), beta blockers, calcium channel blockers, diuretics and other antihypertensive drugs to track new prescriptions of these drugs were recorded. Non-severe hypoglycemia was defined as an event with typical symptoms (e.g., sweating, lose concentration, feeling shaky) disappearing quickly after carbohydrate intake or a status without typical symptoms and plasma glucose ≤ 3.9 mmol/L. Severe hypoglycemia was defined as any episode requiring assistance of another person to recover [9]. Social status was obtained from all participants by a validated questionnaire. Social status, ranging from a minimum of 3 to a maximum of 21 points, was composed of education, highest professional position and household net income [10]. To assess the safety, the following adverse events were documented: dysuria, eGFR below 45 mL/min, urinary tract infection, genital infection, allergic skin reaction, nausea, especially with the aim to identify adverse advents which led to discontinuation of SGLT2 inhibitors, presence of urinary tract—genital infection was assumed if patients reported typical symptoms, or the diagnosis was made by a family physician. Laboratory results were used to confirm the diagnosis if available. If SGLT2 inhibitors were prescribed in our department, all patients were informed about the necessary extra hygienic care. To characterize the effect on antihyperglycemic treatment, we separately analyzed two groups: patients with antihyperglycemic treatment adjustments (hereafter referred to as subgroup 1) and patients without any antihyperglycemic treatment adjustments (hereafter referred to as subgroup 2). We considered it a treatment adjustment if the insulin dose was modified more than 10 percent of the usual dose or an oral antidiabetic drug was added or discontinued. 

### 2.2. Statistical Analysis

All continuous data are shown as mean ± standard deviation (SD). Categorical data are described by absolute and relative frequencies. To compare two groups, unpaired t-test was used for continuous variables and Fisher’s exact test was performed for categorical variables. Paired t-test was used regarding the difference between baseline and follow-up in both groups. Logistic regression models were applied to assess the effect of influencing factors on the discontinuation of the treatment and especially on genital infections. The statistical analysis was performed with SPSS 25.0 (IBM Corp. Released 2017. IBM SPSS Statistics for Windows, Version 25.0. Armonk, NY, USA). Significance was defined at the 0.05 level.

## 3. Results

A total of 170 patients were detected in the patient record. Due to the previously mentioned criteria we excluded 75 patients. The baseline data of the included 95 patients are shown in Table 1. The majority of the observed patients were male (68 males, 27 females). Mean observation time was 1.2 ± 0.8 years.

There was a significantly decrease of HbA_1c_ by −1.0%; 11 mmol/mol (*p* < 0.001), eGFR by −7.0 mL/min (*p* < 0.001), bodyweight by −3.0 kg (*p* < 0.001) as well as BMI by −0.9 kg/m^2^ (*p* < 0.001) and systolic blood pressure by −6 mmHg (*p* < 0.001). The change of diastolic blood pressure (−2 mmHg; *p* = 0.243) and albuminuria (−23.9 mg/g Crea; *p* = 0.189) was not statistically significant. The rate of non-severe hypoglycemia increased insignificantly by 0.01 events per week (*p* = 0.878).

At the beginning the average patient took 1.7 ± 1.2 antihypertensive drugs as long-term medication every day which increased at the end of the observation period to 2.3 ± 1.3 (+0.6 ± 0.8 drugs per patient, *p* < 0.001). Beta blockers were the most prevalent drugs with 0.6 ± 0.5 at the beginning. However, at the end of the trial ACE-inhibitors or ARBs became the most prevalent antihypertensive drug (the mean patient took 0.9 ± 0.3 ACE-inhibitors or ARBs daily).

### Treatment Discontinuation and Adverse Effects

**Figure 1:**
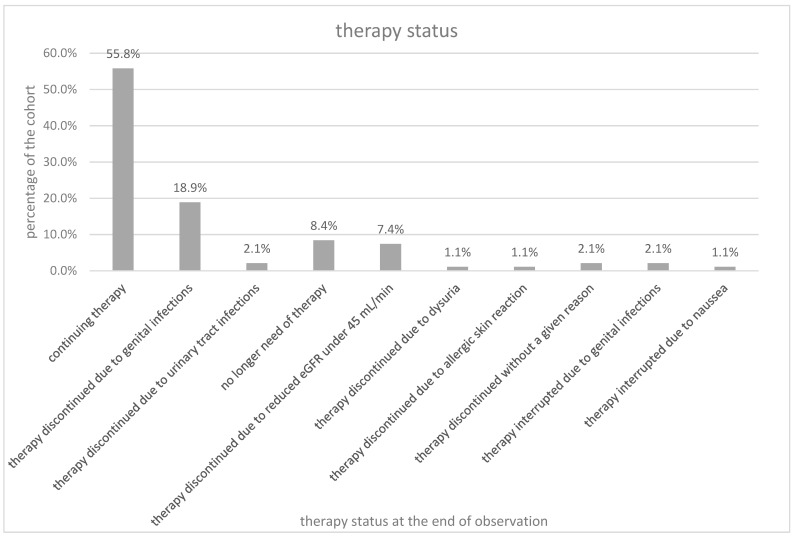
Therapy status.

42 patients (44.2%) discontinued the treatment with SGLT2 inhibitors. The dropouts occurred after a mean observation time of 1.0 ± 0.7 years. Twenty patients (21.1%) discontinued due to genital infections (18 patients—18.9%) or urinary tract infections (2 patients—2.1%). Eight patients (8.4%) no longer needed the therapy because glycemic control allowed to discontinue the medication. Seven patients (7.4%) had a reduced eGFR under 45 mL/min. One patient (1.1%) each discontinued due to dysuria or allergic skin reaction. Two patients (2.1%) quit the treatment without a given reason. Two patients (2.1%) interrupted the therapy due to genital infection and one patient (1.1%) due to nausea but continued the therapy later. The distribution of the mentioned reasons to discontinue therapy are shown in Figure 1. All seven patients who discontinued the treatment because of reduced eGFR under 45 mL/min took either ACE-inhibitors/ARBs (6 participants) or diuretics (1 participant).

We separately analyzed the cohort by gender. The percentages refer to the total count of members of each gender, respectively. Twelve (44.4%) females and 41 (60.3%) males continued the therapy (*p* = 0.120) throughout the observation period. Genital infections occurred in four (14.8%) females and 14 (20.6%) males (*p* = 0.259). Two (2.9%) male participants interrupted the therapy due to a genital infection and continued it later on (*p* = 0.510). Urinary tract infections occurred in two (7.4%) women (*p* = 0.079). The participants who discontinued the therapy due to urinary tract infection or genital infection had a mean age of 64.5 years. The mean age of females in this group was 62.9 years, the mean age of males was 65.2 years. Three (11.1%) women and five (7.4%) men stopped taking SGLT2 inhibitors because the initial reason to start with the medication was not present anymore (*p* = 0.408). The therapy was discontinued because of a reduced eGFR under 45 mL/min in three (11.1%) females and four (5.9%) males (*p* = 0.314). One (3.7%) female patient discontinued SGLT2 inhibitors because of dysuria (*p* = 0.284) and one (3.7%) because of an allergic skin reaction (*p* = 0.284). There was one patient of each gender (1.5% of male patients, 3.7% of female patients) who ended the treatment without a known reason (*p* = 0.490). One (3.7%) male patient interrupted the therapy because of nausea but continued later (*p* = 0.716).

Two models of logistic regressions were performed for discontinuation of therapy. The independent factors of one model were HbA_1c_, BMI, gender, social status and age. In the second model, HbA_1c_, gender, diabetes duration, family status and body weight were the independent factors. In both models, only the higher baseline HbA1c showed a statistically significant association with treatment discontinuations (Model 1: OR = 1.549; CI 1.040–2.306; *p* = 0.031) (Model 2: OR = 1.773; CI 1.112–2.829; *p* = 0.016). The risk of genital infections was lower if ACE-inhibitors/ARBs (OR = 0.136; CI 0.028–0.655; *p* = 0.013) were simultaneously part of the medication, taking into account gender, smoking status, duration of diabetes mellitus and HbA1c, whereas no significant effect was observed for diuretics.

Thirty-seven patients (38.9%) did not have any adjustments in their antihyperglycemic therapy, whereas the therapy of the other 58 patients (61.1%) was adjusted.

Mean HbA_1c_ in both subgroups improved markedly in the observation period and was not different neither at baseline nor at follow up. Also, mean eGFR at baseline and follow up was comparable in both groups. Body weight reduced in both groups but patients of subgroup 1 (with treatment adjustment) lost 1.3 kg less weight than the patients of subgroup 2 (no treatment adjustment) (*p* = 0.307). At the end of the observation subgroup 2 had a 4.9 kg higher mean bodyweight compared to subgroup 1 (105.5 kg vs. 100.6 kg), but this was not statistically significant (*p* = 0.257). Blood pressure in both subgroups differed not statistically significantly in spite of a reduction in systolic blood pressure in subgroup 1.

## 4. Discussion

The purpose of this study was to investigate the effectiveness and safety of SGLT-2-inhibitors in the clinical routine and detect differences between the results of clinical trials and daily healthcare. Our findings showed that SGLT-2-inhibitors were effective as an add-on therapy of diabetes mellitus type 2 treatment. As markers of effectiveness the following parameters were used: HbA1c, eGFR, albuminuria, systolic and diastolic blood pressure, bodyweight and BMI. The HbA1c as parameter for glycemic control showed a significant reduction from baseline to follow-up. eGFR represents renal function and is a common laboratory value. Albuminuria was used as a second renal parameter. SGLT-2-inhibitors are known to be renoprotective [11] but lead to an initial decline of eGFR which stabilizes over time [4]. In our study eGFR decreased on average 7.0 mL/min. To assess if this decline would stabilize, a longer observation period would be necessary. Albuminuria was found to decrease by 23.9 mg/g Crea. Although the change was not statistically significant, a negative trend of albuminuria was observed. Regression of albuminuria is a known effect of SGLT-2-inhibitors and can be considered as a renal outcome [11]. Another well-known effect of SLGT-2-inhibition is an improvement in blood pressure control [12]. The results of our observation showed similar trends. However, there was just a statistically significant reduction of systolic blood pressure, which improved by 5.8 mmHg. Diastolic blood pressure reduced as well, but the reduction of 2 mmHg was not statistically significant due to the small cohort. Nevertheless, a clinically relevant reduction of blood pressure was observed [13]. On the other hand, the number of antihypertensive drugs per patient increased during observation time. Hence, the reduced blood pressure cannot be solely attributed to the SGLT-2-inhibitor therapy and the effect of the altered antihypertensive therapy must be taken into account. Nevertheless, in consideration of the evidence of prospective trials, SGLT-2-inhibitors are certainly adding to blood pressure control in patients with diabetes type 2 [14]. Furthermore, SGLT-2-inhibitors are associated with loss of bodyweight [15]. The patients in this study lost 3 kg over the time of observation and BMI decreased by 0.9 kg/m^2^. At baseline, the observed group had a high bodyweight mean of 105.5 kg and a body mass index of 35.0 kg/m^2^. Obesity is a risk factor for cardiovascular complications and bad outcome for individuals with diabetes mellitus [16], and weight loss for severely overweight people is an important aspect of diabetes care. SGLT-2 inhibitors may be adding to this goal even if the effect is small. 

We divided the cohort into two different subgroups in order to determine the effect if antihyperglycemic treatment was adjusted. Surprisingly, the two subgroups did not show significant differences of treatment parameters. Thus, the influence on the investigated parameters can be attributed to SGLT-2 inhibitors. The development of these parameters also corresponds to those in the literature [6,15]. For a more precise statement, the investigation of a larger cohort would be necessary.

As safety outcome of special interest, we evaluated the discontinuation of therapy due to urinary tract and genital infections. Twenty-one point one percent of study patients discontinued the therapy due to these reasons (18.9% due to genital infections and 2.1% to urinary tract infections), which is far more than in placebo-controlled trials such as EMPAREG with 6.4% of the empagliflozin groups with genital infections [7]. The following reasons could have led to these differing results, compared to controlled trials: We inform all our patients prior to the start with SGLT-2-inhibitors about the possibility of urogenital infections and counsel them to have good intimate hygiene. However, under conditions of clinical routine and outpatient care it is not possible to check for accurate application. Patients attending clinical trials supposedly might follow the recommendations of physicians more strictly. Such patients might be more motivated about being part of a clinical trial. Observation bias (Hawthorne effect) is a possible reason for better hygiene [17]. As previously mentioned, individuals in our cohort had a very high BMI. It is known that obesity is an established risk factor for genital or urinary tract infections [18,19]. Furthermore, patients who discontinued the therapy were older than the average of the whole cohort. High age is a further risk factor for genital and urinary tract infections [20]. Other known risk factors for genital infections are female sex and genital infections occurring before SGLT-2-inhibitor therapy started [18,21]. This information was unfortunately not asked for and thus not available. 

We found that the intake of ACE-inhibitors or ARBs was associated with fewer genital infections and could have a protective effect. The purpose of the study as well as its design did not allow further conclusions on this hypothesis and should be investigated in a prospective randomized trial.

To evaluate the individual risks for future genital or urinary tract infections, the mentioned factors should be considered before initiating SGLT-2-inhibitor therapy. 

Severe hypoglycemia is uncommon with SGLT-2-inhibitors [15]. Thus, we focused on non-severe hypoglycemia. As expected, the rate of non-severe hypoglycemia per week did not increase statistically significantly.

## 5. Conclusions

This study showed that SGLT-2-inhibitors were effective as an add-on therapy in clinical routine of diabetes care. The results were comparable to those of placebo-controlled trials. Glycemic control was improved, presented by a lower HbA1c. There were minor reductions in albuminuria, blood pressure, bodyweight and BMI. eGFR reduced during the first year after initiation of SGLT-2 inhibition. To show long-term stabilization, we would have needed a longer study duration. 

In order to reduce the rate of adverse events of SGLT-2-inhibitors in clinical routine, the mentioned factors should be considered. It is essential to remind the patients to adhere to the hygienic orders. The prescription of SGLT-2-inhibitors should be done on an individual basis taking into regard BMI, sex, age, patient’s history and adherence and ability to follow the hygienic necessities.

## Figures and Tables

**Table 1:** Baseline characteristics of included patients, follow-up data and differences.

	Baseline	Follow-Up	Differences	*p*-Value
Age (years)	60.8 ± 9.7	-	-	-
duration of diabetes type 2 (years)	13.3 ± 8.4	-	-	-
HbA1c (%)	8.9 ± 1.8	7.9 ± 1.2	−1.0 ± 1.9	<0.001
HbA1c (mmol/mol)	74 ± 20	63 ± 14	−11 ± 21	<0.001
eGFR (mL/min)	80.1 ± 18.4	73.1 ± 21.9	−7.0 ± 13.3	<0.001
Albuminuria (mg/g Crea)	164.3 ± 385.5	140.4 ± 387.7	−23.9 ± 144.5	0.118
Bodyweight (kg)	105.5 ± 23.0	102.5 ± 21.8	−3.0 ± 5.8	<0.001
BMI (kg/m^2^)	35.0 ± 6.9	34.1 ± 6.5	−0.9 ± 2.2	<0.001
systolic blood pressure (mmHg)	143.6 ± 22.0	137.8 ± 18.3	−5.8 ± 21.5	0.01
diastolic blood pressure (mmHg)	84.2 ± 13.0	82.6 ± 12.7	−2 ± 13.8	0.243
non-severe hypoglycemia per week	0.06 ± 0.3	0.07 ± 0.4	0.01 ± 0.5	0.878

## Data Availability

The data presented in this study are available on request from the corresponding author. The data are not publicly available due to restrictions of privacy.
